# DNA Nanotechnology-Enabled Fabrication of Metal Nanomorphology

**DOI:** 10.34133/2022/9840131

**Published:** 2022-06-14

**Authors:** Mo Xie, Yang Hu, Jue Yin, Ziwei Zhao, Jing Chen, Jie Chao

**Affiliations:** ^1^State Key Laboratory of Organic Electronics and Information Displays & Jiangsu Key Laboratory for Biosensors, Institute of Advanced Materials (IAM), Jiangsu National Synergetic Innovation Center for Advanced Materials (SICAM), Nanjing University of Posts and Telecommunications, 9 Wenyuan Road, Nanjing 210023, China; ^2^The Interdisciplinary Research Center, Shanghai Synchrotron Radiation Facility, Zhangjiang Laboratory, Shanghai Advanced Research Institute, Chinese Academy of Sciences, Shanghai 201210, China

## Abstract

In recent decades, DNA nanotechnology has grown into a highly innovative and widely established field. DNA nanostructures have extraordinary structural programmability and can accurately organize nanoscale materials, especially in guiding the synthesis of metal nanomaterials, which have unique advantages in controlling the growth morphology of metal nanomaterials. This review started with the evolution in DNA nanotechnology and the types of DNA nanostructures. Next, a DNA-based nanofabrication technology, DNA metallization, was introduced. In this section, we systematically summarized the DNA-oriented synthesis of metal nanostructures with different morphologies and structures. Furthermore, the applications of metal nanostructures constructed from DNA templates in various fields including electronics, catalysis, sensing, and bioimaging were figured out. Finally, the development prospects and challenges of metal nanostructures formed under the morphology control by DNA nanotechnology were discussed.

## 1. Introduction

Metal nanomaterials are preferred by researchers due to their excellent physical and chemical properties (such as small size effect, quantum effect, and surface effect), which depend on their size, shape, chemical composition, and spatial structures [1]. At present, metal nanomaterials have been widely used in the fields of nanoelectronics, photonics, catalysis, and biosensing. However, how to obtain metal nanostructures with designable morphologies and predictable functions based on appropriate manufacturing strategies is still the focus of researchers. Traditional wet chemical [2] synthesis is the most common method. Despite it allows shape control to a certain extent, preparing structures with complex shapes continues to be extremely difficult. Lithography [3] can produce complex structures with high controllable accuracy, but limited by the equipment costs and optical diffraction limits. Therefore, overcoming the problems posed by these traditional manufacturing methods still needs to be constantly explored.

It is an effective strategy to control the morphology of metal nanomaterials through the guidance of templates. Among them, DNA molecule has special potential in template materials used for the assembly of functional materials and the deposition of metal due to the inherent characteristics and advantages of recognition and self-assembly ability, excellent programmability, and abundant chemical functions [4, 5]. With the development of structural DNA nanotechnology, precisely designing DNA nanostructures and using them as templates to deposit metals make shapes of metal transformed from DNA nanostructures possible. By exploiting the addressability of DNA origami, abundant metal morphologies have been prepared, so that the regulation of metal nanomorphologies is not restricted to the shape of DNA nanostructures.

In this review, we first introduce some representative DNA nanostructures and the group features developed by DNA nanotechnology. Then, we review the advances of preparation methods for metal nanomaterials with various morphologies using DNA templates. Furthermore, we briefly discuss the application of metal materials synthesized by DNA metallization strategy in various fields. Finally, we summarize the challenges and prospects of DNA nanotechnology-mediated metal nanomaterials.

## 2. DNA Nanotechnology

**Figure 1:**
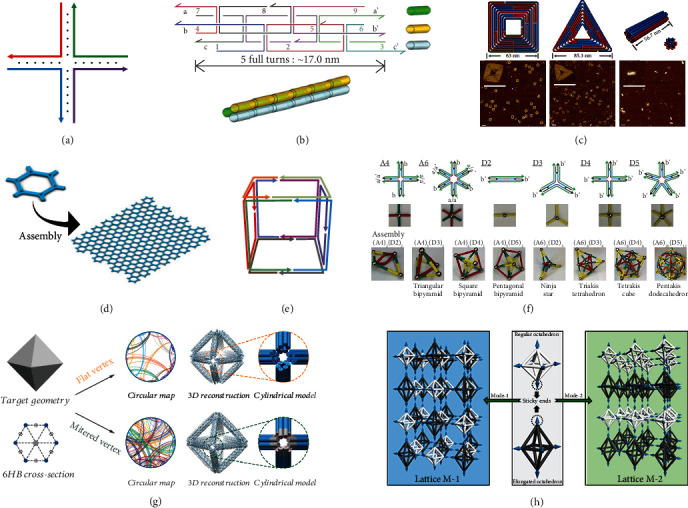
DNA nanostructures. (a) Holliday junction. (b) 3HB tiles arranged into 1D filaments. Reprinted with permission from ref. [10]. (c) 2D DNA origami structures. Reprinted with permission from ref. [19]. (d) DNA origami as building block to construct honeycomb 2D lattices. Reprinted with permission from ref. [21]. (e) DNA cube (each color corresponds to one of ten DNA strands). (f) DNA tiles self-assembly into complex nanocages. Reprinted with permission from ref. [27]. (g) 3D DNA origami structures. Reprinted with permission from ref. [32]. (h) 3D DNA origami lattices. Reprinted with permission from ref. [45].

DNA nanotechnology was first elaborated by Nadrian C. Seeman in the 1980s [6]. He used DNA molecules, which usually considered as carriers of genetic information, as a material component to create nanoscale structures, Holliday junction, which consists of short DNA strands that form a multiarm junction through recognizing specific base pairs (Figure 1(a)). Subsequently, DNA nanotechnology was developed by leaps and bounds; as a building material or combined with other nanomaterials, DNA has brought unprecedented impetus to biology, materialogy, physics, and other fields. Currently, DNA has been used to construct nanostructures of a wide variety of configurations, from simple DNA tile (composed of several short ssDNA of unique sequences with prescribed geometry) [7] and DNA brick (single-stranded tile, SST, assembled from ssDNA as a modular building block) [8] to complex origami (assembled by a long scaffold DNA and hundreds of short staple strands) [9], ranging from one-, two-, to three-dimensional shapes. This section mainly introduces the representative achievements in the construction of DNA nanostructures with various shapes.

### 2.1. 1D DNA Nanostructure

Linear DNA nanostructures have long been promising materials for bottom-up construction of nanoscale conducting wires. Various construction strategies, including tile- and scaffold-based assemblies, for 1D linear structures have been reported to date. In 2005, Park and coworkers proposed a 1D three-helix bundle (3HB) DNA nanostructure consisting of three duplex DNA domains connected by six immobile crossover junctions [10]. 3HB tiles can be further arranged into 1D filaments and 2D lattices by sticky-ends design (Figure 1(b)). Then, they created conductive metal nanowires up to 20 nm in diameter on 1D filaments by metal chemical deposition. In 2013, Ouyang et al. utilized a rolling circle amplification (RCA) strategy to generate long single-stranded DNA (ssDNA) with periodic sequence (96-base) from circular template DNA, which was used as a scaffold to fold into a large nanoribbon structure [11] and used as a template to prepare copper nanoclusters in a later research [12]. Recently, Lat et al. exploited triplex and G-quadruplex hybrid tiles (TQs)*_n_* and assembled a novel reversible composite 1D DNA nanostructure of precise lengths [13].

### 2.2. 2D DNA Nanostructure

The attempt on 2D DNA structure constructions involves assembly based on DNA tiles, DNA bricks, and origami. In 1998, using DNA double-crossover (DX) molecules [14] as basic units, Winfree et al. were the first to construct a 2D crystal structure with periodic stripes up to 2 *μ*m × 8 *μ*m in size [15]. The following year, Mao et al. employed a rhombus DNA structure composed of four Holliday junctions to obtain a 2D periodic array by directional assembly of hydrogen bonds [16]. Afterwards, Yan et al. designed a 4 × 4 tile containing four four-arm junctions which improved stability of Holliday junctions and used for programmable assembly of uniform-width nanoribbons and 2D nanogrid DNA structures [17]; a few years later, they remodeled this 4 × 4 tile using a designing tool named Tiamat [18]. Until 2006, the invention of DNA origami by Rothemund was undoubtedly a landmark achievement in DNA nanotechnology [9]. In his strategy, a 7-8 k long bacteriophage-based single-stranded DNA strand (called “scaffold”) was folded by hundreds of short single-stranded DNA (called “staples”) into the desired 2D shapes (such as square, triangle, pentagram, and smiley face). Since then, other types of DNA origami have been designed (Figure 1(c)) [19], as well as being used as building block to construct infinite or addressable finite size for scaling-up the dimensions. For instance, Woo and Rothemund used blunt-end stacking bonds to connect a finite number of distinct DNA origami [20]. Wang et al. designed a series of hexagonal DNA origami and assembled periodic large-scale 2D honeycomb lattices and tubes through sticky-end connections (Figure 1(d)) [21]. In 2012, Wei group again proposed a series of SST shape designs, which is composed of 42 base ssDNA binds to four local neighbours through sticky ends [22], extending the sequence and material choice. The diversity of 2D DNA nanostructures provides sufficient templates for the formation of multifarious metal morphology. In particular, the addressable DNA origami provides more possibilities for the fabrication of various metal patterns.

### 2.3. 3D DNA Nanostructure

Various construction strategies have been developed for preparing a variety of 3D DNA nanostructures. In 1991, Chen and Seeman reported the first 3D nanostructure named DNA cube. It was a wireframe cube structure formed by the hybridization of ten DNA strands, in which the edges were DNA double helices and the vertices were the branch points of corresponding junctions (Figure 1(e)) [23]. With a similar design, Zhang and Seeman also synthesized DNA-truncated octahedron [24], and Goodman group prepared DNA tetrahedron [25]. Wang and coworkers constructed a variety of DNA polyhedral nanocage structures using tiles with sticky ends as basic building blocks (Figure 1(f)) [26, 27]. After proposing SST [8, 22], Ke group extended the method from 2D to 3D [28]. They constructed more than one hundred complex 3D DNA structures by hundreds of DNA bricks (a canonical DNA brick is a 32-nucleotide single strand with four 8-base sticky ends) in one-step annealing reaction. Shih et al. folded a DNA octahedral frame structure by assembling a 1669-mucleotide single-stranded DNA with five 40-mer strands [29]. This method can be regarded as the prototype of DNA origami technology. The development of DNA origami technology and the development of computer-assisted design software (such as caDNAno [30], DAEDALUS [31], and TALOS [32]) have also provided support for the design of various 3D DNA origami structures. Andersen et al. utilized SARSE software to design a dynamic DNA origami box (a cube structure composed of six DNA sheets) that can be opened by an externally provided DNA key [33]. In the same year, Douglas et al. presented caDNAno software and designed seven 3D rectangular blocks of DNA origami with different cross-section dimensions [30]; using this software, both Sun group and Helmi group designed a series of 3D DNA membranes for the synthesis of 3D gold or silver materials with specified morphologies [34–40]. In addition, a variety of software has been developed to create 3D wireframe structures. In 2015, Benson et al. used vHelix software to render DNA origami 3D polyhedral meshes [41]. Next year, Veneziano et al. created 3D polyhedral network DNA origami structures using the algorithmic framework DAEDALUS [31]. Afterwards, Jun et al. put forward another sequence design algorithm TALOS (Three-dimensional, Algorithmically-generated Library of DNA Origami Shapes) which is also used to fabricate 3D polyhedral wireframe DNA origami (Figure 1(g)) [32]. However, most software could not meet the needs of design and simulation at the same time; Poppleton et al. introduced a web-based visualizer, oxView, that enables basic editing of DNA and RNA and additionally introduced a coarse-grained modelling tool, oxDNA, for simulating DNA/RNA biophysical processes [42]. Furthermore, homogeneous or heterogeneous 2D/3D DNA origami can also be used as building blocks to construct 3D higher-order structures through various assembly strategies. For example, Tikhomirov group used 2D triangular DNA origami for creating a 3D rhombic triacontahedron by design fold symmetry in tile edge, tile concentration, and magnesium [43]. Zhang group designed a 3D triangular strut DNA origami and assembled it into a 3D rhombohedral crystalline lattice through stacking interaction [44]. Ji group synthesized highly ordered DNA crystals by two types of 3D regular octahedron with different symmetries through precisely programming the sticky ends of them (Figure 1(h)) [45]. In 2021, Minev group puts forward a crisscross polymerization strategy; they use multiple ssDNA (conceptualized as a linear array of half- duplex domains) as crisscross slats assemble into 2D and 3D crisscross ribbons (consisting of staggered parallel double helices connected by antiparallel crossovers that occur every half turn) with distinct widths and twists [46]. The successful design and assembly of 3D DNA nanostructures provide an excellent architectural foundation for the synthesis of 3D metallic materials.

## 3. DNA Metallization

The processes of depositing metal nanostructures on DNA template by reducing metal salts are called DNA metallization [47]. Since Braun et al. firstly reported the preparation of silver nanowires by depositing Ag on DNA via chemical reduction in 1998 [48], various methods including chemical reduction, electrochemical deposition [49–51], and photoreduction [52–54] have been developed to produce a variety of metal nanomaterials based on DNA templates. However, no matter which method of metal reduction is adopted, DNA sequences or DNA nanostructures can be precisely designed and used as templates to guide the formation of metal nanostructures, so that their morphology can be determined. In this part, we will focus on the existing strategies of using DNA template to manufacture various metal nanostructures with controllable shapes, including nanoparticles, nanowires, and nanopatterns.

### 3.1. Nanoparticle

DNA-mediated synthesis of metal particles, including smaller clusters and larger particles, has properties that are rest with size and shape. Since the growth morphology of metal nanoparticles is DNA template dependent, in order to prepare nanoparticles with adjustable functions, it is necessary to clarify the DNA sequence or structural template used to form the nanoparticles. In this section, we will summarize the growth regulation of various nanoparticles based on DNA templates.

#### 3.1.1. Clusters

**Figure 2:**
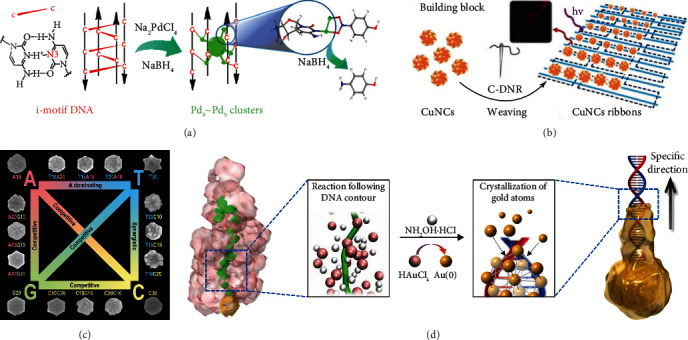
DNA-mediated synthesis of metal nanoparticles. (a) i-motif DNA as templates for subnano-Pd clusters synthesized. Reprinted with permission from ref. [57]. (b) DNA nanoribbons as templates for ultrasmall copper nanoclusters (CuNCs) synthesis and assembly. Reprinted with permission from ref. [12]. (c) DNA sequences regulate gold nanoparticle morphological evolution. Reprinted with permission from ref. [62]. (d) dsDNA-directed the growth of gold nanocrystals. Reprinted with permission from ref. [66].

Metal nanoclusters are composed of a few to hundreds of metal atoms, and adjusting their size is significance to electronic, optical, and catalytic properties. The growth of clusters could be controlled by the spatial constraints provided by DNA structure templates. Single-strand DNA and its secondary structures are the most commonly used templates [55–57]. Zhang et al. utilized i-motif DNA with consecutive hemiprotonated cytosine-cytosine (CH^+^·C) as template, in which Pd ion precursors can effectively bind to the N3 sites of cytosines, then catalytically active subnano palladium clusters with determined number of Pd atoms generated through controlling the [Pd]/[base] ratio and further NaBH_4_ reduction (Figure 2(a)) [57]. Afterwards, double-strand DNA was also used as template for prepare clusters. For the first time, Park et al. used branched double strand DNA (x-shaped DNA and y-shaped DNA) as scaffold to synthesize brightly fluorescent silver nanoclusters (AgNCs) [58]. Among them, X-DNA produced brighter AgNC with the photoluminescence quantum efficiency of nearly 20%. Additionally, DNA nanostructure templates also show excellent potential in proving the photostability and adjusting the excitation/emission properties of metal clusters [12, 59]. Ouyang et al. employed DNA nanoribbons containing multiple specific binding sites as templates for ultrasmall copper nanoclusters (CuNCs) synthesis and assembly in situ [12]. CuNCs arranged tightly and orderly on the nanoribbons, which enhanced the luminescence performance and fluorescence stability (Figure 2(b)).

#### 3.1.2. Particles

DNA exhibits sequence-dependent affinity for silver, gold, and other nanoparticles through the interaction of nitrogen and oxygen on nucleobases and sugar groups as well as the electrostatic interactions of phosphoric backbones, which makes DNA easily combined with nanoparticles [60, 61]. What is more, DNA sequences anchored on the seeds have significant effects on the morphology of metal particle formation in seed-mediated synthesis methods.

Researchers confirmed that the influence of different ssDNA sequences on the morphology evolution of metal nanoparticles grown from different seeds. For the morphology regulation of gold nanoparticles (AuNPs), Wang group has conducted a great deal of research in this regard. In 2012, they proposed the role of different DNA sequences and their combinations in controlling the growth of gold nanoprism seeds and summarized the rules of the evolution of nanoseeds into different shapes according to the DNA sequences (Figure 2(c)) [62]. When using ssDNA that contains one type of deoxyribonucleotide, nanoparticles with A30 grew into round nanoplates with rough surfaces, while formed six-pointed nanostars with T30, nanoparticles with C30 yielded round nanoplates with smooth surfaces, and G20 synthesized hexagonal nanoplates. When DNA contains two types of deoxyribonucleotide, competitive and synergistic effects were observed. For the morphology regulation of silver nanoparticles (AgNPs), Li group did research on DNA-mediated growth of spherical Ag seeds [63]. Poly C and poly G leads growth to nanoprisms, while poly A and poly T lead growth to flower bouquets and nanodiscs, respectively. Besides, they found that the length of DNA had little effect on the morphology of AgNPs. Subsequently, ssDNA sequences regulate the form of other shapes of metal nanoparticles (i.e., gold nanoflowers [64]), and bimetallic nanoparticles (i.e., Pd-Au bimetallic nanostructures [65]) have been reported.

In addition to ssDNA, double-stranded DNA (dsDNA) also applied to direct the growth morphology of metal nanostructures. Ma et al. put forward a method for preparing asymmetric Au nanoparticles [66]. They used dsDNA as molecular regulator anchored onto gold nanoseeds (AuNS) for drive the growth of gold nanocrystals to desired shape (including asymmetric pushpin-, water caltrop-, ‘T'-like shapes and other complex structures). Simulation showed that redox reactions mostly happened at the dsDNA/AuNS conjugation region, since the reducing agent NH_3_OH^+^ was mainly around dsDNA and AuCl_4_^−^ was mainly in a rather distance space, while AuNS lowered the activation energy of reduction (Figure 2(d)). Furthermore, low-molecular weight PEG on seeds promotes the impart directionality of dsDNA during crystallization. Recently, they further utilized dsDNA for direct gold crystallization to synthesize rod-like nanoparticle with bridges through the similar strategy. Adjusting the length of dsDNA and surface charges could subtly adjust the nanobridges or nanogaps of plasmonic gold nanoparticles for biosensing [67].

### 3.2. Nanowire

It is undoubtedly unfortunate that most of the electronic properties of bare DNA are insulating limiting its application in electronics [68]. However, the proposal of DNA metallization overcomes the intrinsic low conductivity of DNA and allows conductive wires to be constructed in nanoscale [48]. In this section, we will introduce the established strategies for various types of metal nanowires, including continuous, segmented, and branching nanowires.

#### 3.2.1. Continuous Nanowires

**Figure 3:**
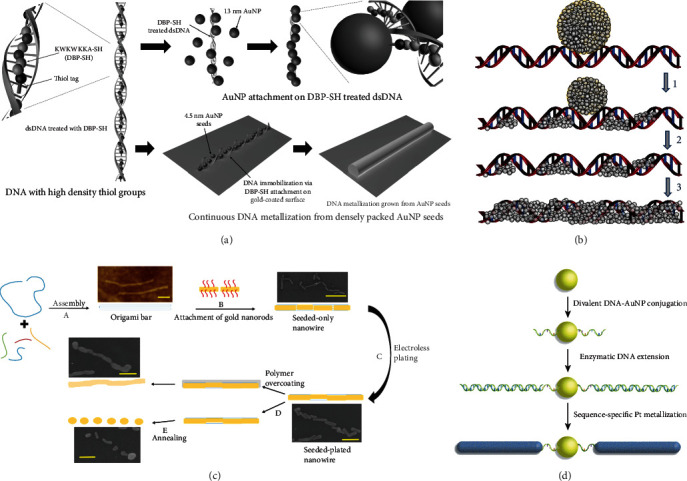
Metallic nanowires on DNA templates. (a) Synthesis of continuous DNA nanowires using thiol-tagged DNA-binding peptide (KWKWKKA-SH, DBP-SH). Reprinted with permission from ref. [70]. (b) The transformation process of dsDNA to E-DNA. Reprinted with permission from ref. [71]. (c) Preparation of DNA origami-templated gold nanowires and the process of polymer-constrained annealing. Reprinted with permission from ref. [76]. (d) The metallization of divalent DNA-nanoparticle conjugates for fabricate SEDs. Reprinted with permission from ref. [78].

At present, metal nanowire fabrication process involves activated by attaching metal ions or seeds on DNA backbone and then can be further deposited and grown [69]. Since smooth and continuous structure of metal nanowire is beneficial to improve the electrical conductivity, researchers have put forward various effective strategies to improve the preparation. Kim et al. treated dsDNA with thiol-tagged DNA-binding peptide (DBP-SH: KWKWKKA-SH) to introduce a large number of thiol functional groups. Then, the peptide-bound DNA molecules were elongated and attached on gold-coated surface by thiol-gold interaction. Next, 13 nm AuNPs as seeds were densely immobilized on dsDNA backbone via thiol groups of DBP-SH and ultimately guiding smooth and continuous DNA metallization without branching (Figure 3(a)) [70]. Eidelshtein et al. proposed a novel preparation method of silver-containing DNA molecules (E-DNA, E stands for electrical) [71]. AgNP attaches Poly(dG)-Poly(dC) DNA and donates Ag atoms to nucleic acid, resulting in silver atoms and a few atomic clusters being located on or inside the DNA molecule (note that this step is sequence selective due to higher affinity of G and C bases to Ag ions). After AgNP dissociated and binding to DNA, Ag atoms obtained electrons from the reduced guanine radical, and finally E-DNA produced by a massive binding-dissociation cycles (Figure 3(b)). Moreover, they improved the conventional deposition process after seeding AuNPs in two ways [72]. On one hand, gold salt and ascorbic acid were mixed directly to form enhanced solution for reducing the possibility of self-seeding in solution. On another hand, the concentration of gold salt in enhanced solution was changed in order to control the growth of gold layer thickness.

#### 3.2.2. Segmented Nanowires

Controlled wiring is necessary in preparing electronic circuits with complex functions, which requires positioning and manufacturing in molecular precision. After propose to fabricate continuous conductive nanowires with DNA templates [48], Keren group once again proposed a sequence-specific molecular lithography technology to fabricate segmented gold wires on single DNA molecules [73]. Taking advantage of assembly and resist properties of RecA protein, the nucleoprotein filaments formed by RecA polymerized on the ssDNA probe molecules were combined with aldehyde-derivatized dsDNA substrates via homologous recombination for sequence-specific metal patterning. Ag aggregates were formed in the RecA unprotected region of dsDNA after incubation in AgNO_3_, thereby catalyzed gold deposition and in situ formation of conductive gold wires. Based on this technology, they realized the precise localization of carbon nanotubes (SWNT) through a three-strand homologous recombination reaction that nucleoprotein filaments guided SWNT to specific locations on dsDNA molecule, realized the fabrication of carbon nanotube field-effect transistor (SWNT-FET) [74]. Next year, they further improved the technology. Following homologous recombination, incubation with glutaraldehyde resulted in aldehyde-derivatization of dsDNA in regions without RecA protected, which was beneficial for preserving the biological function of the DNA molecule; subsequent specific reduction of silver ions occurred in the aldehyde-derived region and formed segmented silver clusters [75]. Recently, Westover et al. developed a new method to control the morphology and conductance of nanowires through annealing temperatures [76]. Nanowires were formed by electroless plating gold on gold nanorod-seeded DNA origami structures. At low annealing temperature exceeding 180°C, plated nanowires broke up to form isolated islands (Figure 3(c)).

#### 3.2.3. Branched Nanowires

It is well known that there is not only one path in electronic circuit, but multiple branches. Accordingly, manufacture of branching nanowires is necessary. Currently, branched metal nanowires could be obtained through branching nanostructure templates, which were mainly incorporating central nanoparticles (AuNP, streptavidin, biotin-DNA complex, etc.), branching units (DNA molecules), and bridging interaction (DNA hybridization, conjugation, specific affinity, etc.) [77–79]. For example, Wang et al. proposed a method for constructed single-electron devices (SEDs) by metallization of divalent DNA-nanoparticle conjugates [78]. The DNA conjugated to AuNPs consisted of an ssDNA portion and an enzymatically extended dsDNA portion. Subsequent sequence-specific Pt metallization formed conductive Pt nanowire structures (Figure 3(d)). Rudiuk et al. used monobiotinylated DNA branches attached to streptavidin to constructed branched templates, the length of branches was regulated by the number of DNA bases, and the degree of branching was controlled by the concentrations of the two components [79]. Silver nitrate and reducing agent NaBH_4_ were added to metallizing the templates to obtain branching silver nanowires.

### 3.3. Nanopattern

Complex functional devices or functional systems, such as sensors, nanoelectronic devices, and surface plasmon-based circuits, rely on the fabrication of metal pattern structures with nanometer resolution. DNA template is a powerful tool for guiding the assembly and synthesis of metals into specific patterns with high complexity. In this section, we will document various methods of DNA templated-mediated metal pattern preparation.

#### 3.3.1. Nonselective Templates Regulate Metal Nanopattern

**Figure 4:**
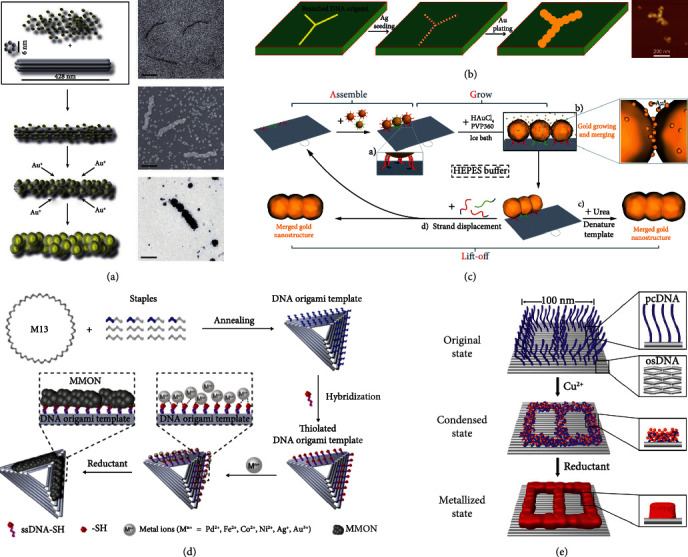
DNA nanostructures as templates to regulate metal nanopattern. (a) DNA origami structures preseeded by electrostatic interactions serve as templates for nonselective metallization. Reprinted with permission from ref. [80]. (b) Ag preseeding on aldehyde group-modified DNA origami structures as templates for nonselective metallization. Reprinted with permission from ref. [81]. (c) AGLO strategy to construct a predesigned gold nanostructure. Reprinted with permission from ref. [89]. (d) Thiol groups arranged DNA origami for precise organization of metal and metal oxide nanoclusters. Reprinted with permission from ref. [95]. (e) DCIMP strategy for selective metallization. Reprinted with permission from ref. [97].

Programming the DNA nanostructure template with defined shape, metal grows to cover the templates until a copied metal pattern formed. Among them, templates seeded with small nanoparticles and then plated with metal via electroless deposition process is the most common method for metal growth. So far, researchers have proposed several seeding and growing techniques. Schreiber et al. adopted the principle of electrostatic interaction of positive and negative objects to seeding; they introduced 1.4 nm Au clusters coated with positively charged amines to negatively charged DNA origami of different shapes as seeding sites for gold ion deposition and further grown into continuously gold nanostructures via an electroless deposition process (Figure 4(a)) [80]. Various metallized structures of arbitrary shapes have been prepared by using this process. Liu group presented several prereduction processes for seeding. In 2011, this seeding process was implemented by reducing silver ions with aldehyde group-modified DNA origami [81]. Firstly, amine-modified psoralen was crosslinked to DNA origami and purified by dialysis. Secondly, the purified origami was reacted with glutaraldehyde to obtain aldehyde group-modified DNA origami and purified again. Thirdly, silver ions are added to initiate seeding. And finally, added gold plating solution to yield T- or Y-shaped gold nanostructure (Figure 4(b)). In the same year, they put forward another seeding process that reduced Pd ions coordinated with N atoms on nucleobases [82]. In this research, three steps including Pd activation, reduction to form seeds, and electroless plating were utilized to achieve DNA metallization to form Pd and Au nanostructure. In 2013, Geng group used the same process again to prepared circuit-like Cu and Au nanostructures [83]. In addition, researchers also used DNA hybridization strategy to seeding the DNA origami, that is, assembling DNA-modified AuNPs to DNA origami. AuNPs as seeds for growing enhanced gold nanoparticles until fused with each other [84, 85].

#### 3.3.2. Selective Templates Regulate Metal Nanopattern

Metallic nanostructures present extraordinary potential for photonics and electronic, and such construction requires precise control for the position of the metal at nanometer resolution. In fact, site-specific DNA metallization strategies allow the selective growth of metal in a defined location of DNA templates. In particular, addressable DNA origami has shown great preponderance in this selective template regulation. Currently, precise manufacturing of metal nanopattern on DNA nanostructure template mainly involve the following three strategies.

Hybridization of ssDNA-modified nanoparticles with complementary sequences extended at design positions on DNA nanostructures that provide nucleation sites for metal growth to obtain nanopattern is a relatively common method. One of the most commonly used seeds are gold nanoparticles [86–90]. For example, Luo et al. came up with a grow and lift-off (AGLO) strategy [89], that is, initial association of AuNP seeds to the binding sites of DNA origami, then Au slowly deposited on the seeds, allowing the seeds to grow until it merges with the neighbors and forms the predesigned shape and finally lift off the origami from it (Figure 4(c)). Gold nanorods are also used as seeds. Based on gold nanorod seeding and metal deposition processes, various continuous metal 2D geometric shapes (rectangular, square, and T shapes) with as small as 10 nm in diameter were prepared by Uprety group [91]. Moreover, Aryal et al. reported for the first time that multiple metal junctions can be produced by seeding gold and tellurium nanorods in stages and electroless plating [92].

Site-specific arrangement of reducing groups on DNA nanostructures is another effective strategy to form precise metal patterns. Pal et al. covalently incorporated sugar moieties into staple strands at adjacent positions of DNA origami [93]. Tollens reactions enabled the Ag ions reduce to AgNCs that could act as seeds for further Ag deposition in situ. Roy et al. reported a 2′-deoxyoligonucleotides containing boranephosphonate linkages (bpDNA), which can assemble DNA nanostructures via Waltson-Crick base pairs. The bpDNA allowed the metal ions (AuCl_4_^−^, Ag^+^, and PtCl_4_^2−^) to be site-specifically reduced to corresponding nanoparticles on DNA nanostructure [94]. In recent studies, thiol groups initiated metal reduction was also used to achieve site-selective metallization [95, 96]. For instance, Li group reported an in situ metal and metal oxide nanoclusters (MMONs) growth approach [95], which expressly arranged thiol groups on DNA origami and used them as reaction sites to grow MMONs (Figure 4(d)). They extended the approach to synthesis of palladium, cobalt, nickel, silver, gold, and Fe_2_O_3_ nanoclusters with arbitrary patterns, which proved the universality of this strategy.

Interaction between metal precursor ions and bases allows directly growing of metal nanoparticles on DNA nanostructures, but most of this method lacks addressability. However, Jia group proposed a highly localized metallization reaction with DNA origami for metal patterns with 10 nm resolution called DNA condensation and intrinsic metallization patterning (DCIMP) [97]. They have demonstrated that strong coordinate between low-valence metal ions (Cu^2+^ and Ag^+^) and DNA bases in flexible protruding-clustered DNA (pcDNA) prescribed on 2D DNA origami leads to pcDNA condensation, and then, the selectively metallization reaction occurs on pcDNA instead of on the dsDNA-based origami substrate (osDNA) at appropriate metal concentrations (Figure 4(e)). Recently, Zhang group extended this strategy to the three-dimensional chirality of silver nanostructure preparation in aqueous solution [98]. In their study, diamine silver(I) complexes were found to coordinate with pcDNA on tubular DNA origami via coordination, hydrogen bonds, and ion-*π* synergetic interactions and induced local enrichment of silver precursors on pcDNA, reducing the activation energy of nucleation. They further demonstrated the customizable plasmonic optical activity of metallized chiral silver nanostructures. This metallization strategy opens up a new way to synthesize programmable 2D and 3D inorganic materials with arbitrary morphologies and properties.

#### 3.3.3. Mold Regulation

**Figure 5:**
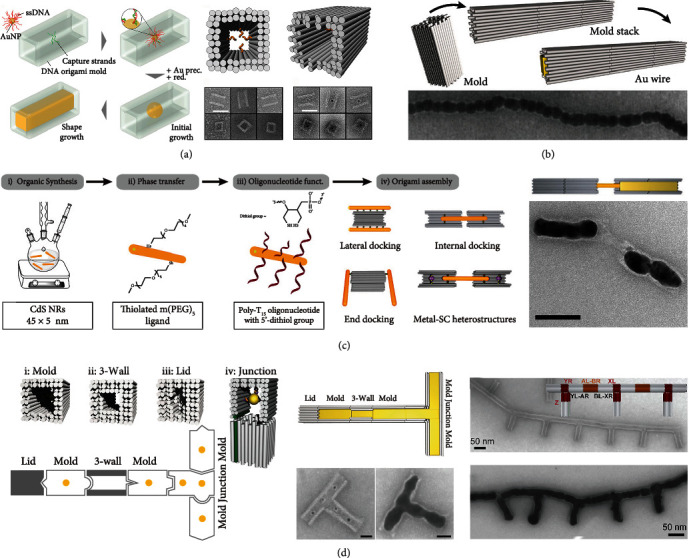
DNA molds for regulation of metal structure construction. (a) DNA molds constrain the growth of gold nanostructures. Reprinted with permission from ref. [35]. (b) DNA mold bricks assembled line structures for prepare conductive gold nanowires. Reprinted with permission from ref. [40]. (c) The assembly of DNA mode and semiconductor nanorods (SC NRs), and molds regulate metal growth. Reprinted with permission from ref. [39]. (d) DNA origami mold superstructures for complex metal nanostructures synthesis. Reprinted with permission from ref. [36].

Mold regulation strategy mainly is carried out by using DNA nanomolds with 3D cavities to guide the metal nanostructures growth into desired shapes. Sun group proposed this strategy almost simultaneously in 2014 [34]. In Sun et al.'s work, they designed and assembled a rigid DNA nanostructure mold that contained a DNA mold with lids, a 3D cavity with a nucleating gold “seed” inside, and the seed grew into a larger metal structure until fills the cavity under mild conditions [34, 35]. According to this method, they synthesized a variety of nanoparticles with a three-nanometer resolution. Compared with Yin's work, the 3D cavity molds without lids were used in Helmi et al.'s work [35], and higher-order molds were also used to grow gold particle dimers. Besides, they found that the final growth size of gold nanoparticles in the cavity was controlled by the ratio of HAuCl_4_ to AuNP seed. The seed grown in the cavity and then expands along the shape of the cavity until HAuCl_4_ were consumed (Figure 5(a)). In follow-up work, Bayrak group extended this approach. In 2018, individual molds were assembled into micron-sized mold superstructures; AuNP seeds inside were grown to prepare conductive gold nanowires (Figure 5(b)) [40], while the growth conditions and procedures of gold seeds were studied in detail in 2020 [37]. In 2019, they accurately adjusted the interfaces between the molds that enabled each mold monomer remains uniquely addressable, then used it to explore the site-specific metallization and created defined metal pattern [38]. In the same year, they presented a new CdS nanorod oligonucleotide functionalization method to realize the assembly of DNA origami and semiconductor nanorods (SC NRs) and again extended the approach to the preparation of semiconductor nanorods (Figure 5(c)) [39]. Recently, Ye group further improved their mold-based fabrication strategy, and successfully established a versatile platform for modular and programmable manufacturing of gold nanostructures [36]. They developed and synthesized more complex gold nanostructures (including tightly DNA-caged particles, rolling-pin- and dumbbell-shaped particles, T-shaped, L-shaped, and loop particles) via designing and assembling individual building blocks to construct DNA origami mold superstructures of different predetermined geometries (Figure 5(d)). These reports could pave the way for future manufacturing 2D or 3D circuit structures.

#### 3.3.4. DNA as Mask for Nanolithography

The mentioned above are all bottom-up-based approaches of transferring the spatial information of DNA structures to metal structures; most of them are directly synthesized on templates or chemical growth of attached seeds. Nevertheless, a combination of bottom-up (molecular self-assembly) and top-down (common lithography) is also an excellent method for controlling metal morphology, that is, using DNA nanostructure as lithography mask.

**Figure 6:**
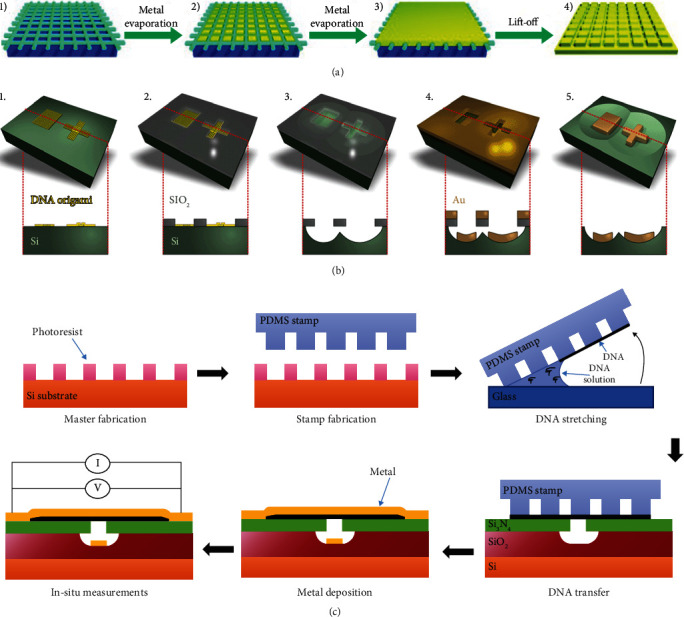
DNA as mask for nanolithography. (a) Process of DNA molecular lithography. Reprinted with permission from ref. [99]. (b) Metallic structures preparation based on DNA origami nanoshape silhouettes on silicon substrates. Reprinted with permission from ref. [100]. (c) DNA templates precisely control metal nanowire fabrication via microcontact printing. Reprinted with permission from ref. [103].

In 2004, Deng and Mao demonstrated that DNA nanostructures can be used as molecular masks to prepare nanoscale metal patterns [99]. They achieved this molecular lithography in four steps: (1) depositing DNA nanostructures on mica substrates; (2) and (3) evaporating metal onto the mica substrates and covering the DNA mask until a continuous metal film was formed, then add the epoxy mixture to solidify between the gold film and the glass slide at the end of the evaporation; (4) removing the glass slide together with the metal film from the DNA/mica surface to obtain the 1D and 2D metallic nanopatterns (Figure 6(a)). In order to create custom-shaped metal nanostructures with higher precision, Shen et al. reported a DNA-assisted lithography (DALI) technique [100], which combined the chemical vapor deposition (CVD), lithography, wet etching, and DNA nanostructure. Firstly, DNA origamis were deposition on the Si substrate. Then, a SiO_2_ layer was selectively grown on the Si substrate without DNA origami by CVD process, and DNA origami-shaped silhouettes were created, which allowed the underneath Si layer etched away by reactive ion etched (RIE). Next, metal was deposited on the chip by electron beam evaporator in ultrahigh vacuum. Finally, the SiO_2_ layer and the metal on top were removed, and the metal nanostructures with DNA origami morphology were obtained (Figure 6(b)). Later, they prepared complex metal nanostructures with optical properties through this DALI method [101]. Although DALI technique is indeed a competitive metal nanomanufacturing method, it relies on hydrofluoric acid (HF), which makes it unsuitable for some masks, metallic materials, and transparent substrate materials. In 2020, Piskunen and coworkers presented a new technique called biotemplated lithography of inorganic nanostructures (BLIN) [102] to circumvent the limitations of DALI. They expanded the masks, not only using DNA origami but also Tobacco mosaic virus (TMV) as pattern templates. Moreover, they overcome the material limitations; patterned Au, Ag, Al, Ti, and Ge were fabricated on Si and ITO glass substrates. Furthermore, a sacrificial acetone-removable layer was added between the substrate and grown Si layer to avoid undesired HF etching.

However, in the above strategies, DNA templates were randomly assembled, while the number, orientation, and location of the metal nanostructures could not be precisely controlled. In 2020, Barreda et al. demonstrated a process for precise placement and preparation of nanowires [103]. By dewetting and stretching the DNA molecules, DNA nanowires were formed on the micropillar array of polydimethylsiloxane (PDMS) with a designed size and spacing. Then, the DNA nanowires were transferred from the PDMS stamp to a prefabricated substrate (Si/SiO_2_/Si_3_N_4_) by microcontact printing. Metal deposited onto substrate and crossed the trench with a Si_3_N_4_ overhang to form the continuous metal nanowires at 0.9 nm thickness with a cross-section of few nm^2^ (Figure 6(c)). These researches have expanded the controllability of DNA in the manufacture of metal nanostructures.

## 4. Applications

Based on DNA nanotechnology and DNA template, DNA metallization technology indeed provides a simple and flexible method for guiding the synthesis of metal nanomaterials of different types, morphologies, and properties. The synthesized metal nanomaterials have shown great valuable application in nanoelectronics, catalysis, sensing, bioimaging, and other fields due to their composition, size, and morphology-related physicochemical properties. The representative researches in each field are briefly introduced below.

### 4.1. Electronics

One of the most important applications of DNA metallization research is to help realize the positioning and conducting connections between nanounits. Overcoming related technical problems through a bottom-up strategy is of great value for the construction of functional nanoelectronic devices and circuits. In 2003, Keren et al. used DNA scaffold molecules as templates to precisely locate semiconductor single-walled carbon nanotubes and extend the metal wires in contact with them, achieving a self-assembled carbon nanotube field-effect transistor capable of operating at room temperature [74]. To address the issues of irregularity and lack of end-to-end electrical connectivity in metallized DNA nanowires, Stern and coworker reported a narrow gold-coated DNA wire with improved smoothness and uniformity, as well as breakthroughs in long-range conductivity; this was the first report of distance 150 nm for a wire, only 10 nm high, and 400-700 nm for a wire as low as 13 nm [72].

### 4.2. Catalysis

Recently, metal nanomaterials have attracted much attention due to their high surface area, selectivity, adjustable morphology, and remarkable catalytic activity which depend on the species, size, shape, and surface area to volume ratio of metal nanomaterials determining the activity of metal catalyst [104]. DNA metallization provides a flexible and tunable method to prepare metal catalysts with different properties. In the past few years, DNA-based metal nanoparticles have been widely used as catalysts. For example, Zinchenko et al. used DNA cross-linked hydrogels as a matrix for the synthesis of well dispersed, nonaggregated gold nanoparticles with 2-3 nm size [105]. Afterwards, they again prepared various transition metal nanoparticles (Au, Ag, Pt, Pd, Cu, and Ni) with 2-3 nm size and investigated the catalytic activity of metallized hydrogels for the reduction of nitroaromatic compounds. The results showed that the catalytic activity as follows: Pd > Ag ≈ Au ≈ Cu > Ni > Pt [106]. Since the interaction mechanism between DNA template and metal ions affects the physicochemical properties of nanoclusters [57, 107], Fu et al. regulated the physicochemical properties (including charge state and particle size) of Pt nanozymes by programming the DNA template sequence and controlling the precursor ions and the molar ratio of [precursor]/[DNA]; this nanozymes are showing high activity against peroxidase [108].

### 4.3. Sensing

One of the methods to construct sensors is to exploit the electrochemical activity of metallized materials. Hao et al. constructed an ultrasensitive electrochemical biosensor for the rapid detection of CYFRA 21-1 DNA (target DNA, tDNA) [109]. DNA metallization by surface-initiated reversible addition fragmentation strand transfer (SI-RAFT) polymerization facilitates amplification of the detection signal, and the detection limit can be as low as 0.89 aM. Wu et al. presented an ultrasensitive electrochemical telomerase activity-sensing strategy, which was the first attempt to detect telomerase activity in circulating tumor cells (CTCs) by using PCR-free method [110]. In their study, DNA template-deposited silver nanoparticles were utilized for signal amplification, and telomerase activity in CTCs was detected by enzyme-assisted background current-suppression and highly characteristic solid-state electrochemical process. In addition to DNA and enzymes, DNA-metallized materials can also be used to detect heavy metal ions, such as Hg^2+^, Pb^2+^, and Cu^2+^ [111–114]. Furthermore, DNA metallization technology has also been used to produce various sensors for detecting gases, including hydrogen sensors [115], ammonia sensors [116], and humidity sensors [117].

### 4.4. Bioimaging

The appearance of metal clusters with the advantages of small size and good brightness has opened up a path for biolabeling. Among them, DNA-templated silver nanoclusters (AgNCs) can be used for highly sensitive biomarkers owing to it can be easily synthesized and tunable emission color. Lim et al. investigated the effect of four basic DNA nucleotides on AgNC synthesis and its photoluminescence properties and established empirical design rules; by rationally modifying the DNA template sequence to control its luminescence color and biological function, the application of multicolor targeted bioimaging could be realized [118]. However, the native D-conformation of DNA (D-DNA) is highly sensitive to nucleases, Han et al. demonstrated that L-conformation of DNA (L-DNA), the enantiomer of D-DNA, can also be used to synthesize silver nanoclusters (AgNCs) with bright fluorescence. Moreover, compared with AgNCs synthesized from D-DNA templates, AgNCs synthesized from L-DNA templates have higher nuclease resistance than those synthesized from D-DNA templates, which makes them useful as biostable optical probes for cell identification and imaging [119].

## 5. Summary and Outlook

DNA nanotechnology has made tremendous advances in the past decades. The construction of DNA nanostructures has experienced from the design of the primitive to the assembly of 1D, 2D, and 3D structures; meanwhile, DNA nanotechnology and DNA nanostructures have also been used in the assembly and synthesis of various metal materials. Among them, DNA-templated metallized nanomaterials have unique advantages in the regulation of metal nanoforms, which cannot be achieved by other traditional synthetic methods.

In spite of various methods have been developed to fabricate metal nanomaterials with specific morphologies and compositions on DNA templates, challenges still remain: (1) the detailed mechanism and ultimate function of DNA-mediated growth is still poorly understood. This still requires synergy between DNA metallization and multiple fields to establish a scientific connection between theoretical and experimental foundations. (2) For metal materials that need to be synthesized in complex chemical environments (such as extremely acidic or basic conditions), the stability of DNA nanostructure templates needs to be improved under such conditions. Furthermore, it is necessary to regulate the species and components of metals to synthesize polymetallic materials, which is of great significance to further expand the types of metal materials with controllable morphology. (3) The shape and size of metal nanostructures are key influencing factors for their properties; however, the presence of nonspecific nucleation during metal synthesis often interferes with the expected structure and function. This requires an in-depth exploration of the kinetics and thermodynamics of metal nucleation and growth. (4) DNA-based metal nanoparticles still face problems of poor biocompatibility and stability, which are related to the release of heavy metals from nanostructures. Therefore, it is necessary to investigate the ultimate fate of various nanostructured elements in the body and to use the chemical function of DNA to prepare new materials without toxic side effects. (5) DNA origami as a template for creating patterned metals is a very promising method for electronic device manufacturing. However, the uniformity and continuity of the grown metal materials still need to be improved. In addition, the expansion of structure size and large-scale production still require the promotion of the development of DNA nanotechnology. To sum up, improving the synthesis process and preparing high-quality metal nanomaterials with high hand applicability is still the focus of this research direction. We also believe that with the continuous improvement of existing technology, it is foreseeable that DNA metallization will have greater progress and breakthrough in terms of more complete synthesis and practical applications in the future.

## References

[1] Zhou M., Li C., Fang J. Y. (2021). Noble-metal based random alloy and intermetallic nanocrystals: syntheses and applications. *Chemical Reviews*.

[2] Ahmed S., Annu S. I., Yudha S. S. (2016). Biosynthesis of gold nanoparticles: a green approach. *Journal of Photochemistry and Photobiology B: Biology*.

[3] Wang Y. Y., Fedin I., Zhang H., Talapin D. V. (2017). Direct optical lithography of functional inorganic nanomaterials. *Science*.

[4] Zhao Y., Dai X. P., Wang F., Zhang X., Fan C., Liu X. (2019). Nanofabrication based on DNA nanotechnology. *Nano Today*.

[5] Li N., Shang Y., Han Z., Wang T., Wang Z. G., Ding B. (2019). Fabrication of metal nanostructures on DNA templates. *ACS Applied Materials & Interfaces*.

[6] Seeman N. C. (1982). Nucleic acid junctions and lattices. *Journal of Theoretical Biology*.

[7] Wang P. F., Chatterjee G., Yan H. (2017). Practical aspects of structural and dynamic DNA nanotechnology. *MRS Bulletin*.

[8] Yin P., Hariadi R. F., Sahu S. (2008). Programming DNA tube circumferences. *Science*.

[9] Rothemund P. W. (2006). Folding DNA to create nanoscale shapes and patterns. *Nature*.

[10] Park S. H., Barish R., Li H. Y. (2005). Three-helix bundle DNA tiles self-assemble into 2D lattice or 1D templates for silver nanowires. *Nano Letters*.

[11] Ouyang X., Li J., Liu H. (2013). Rolling circle amplification-based DNA origami nanostructrures for intracellular delivery of immunostimulatory drugs. *Small*.

[12] Ouyang X., Wang M., Guo L. (2020). DNA nanoribbon-templated self-assembly of ultrasmall fluorescent copper nanoclusters with enhanced luminescence. *Angewandte Chemie International Edition*.

[13] Lat P. K., Schultz C. W., Yu H. Z., Sen D. (2021). A long and reversibly self-assembling 1D DNA nanostructure built from triplex and quadruplex hybrid tiles. *Angewandte Chemie International Edition*.

[14] Fu T. J., Seeman N. C. (1993). DNA double-crossover molecules. *Biochemistry*.

[15] Winfree E., Liu F., Wenzler L. A., Seeman N. C. (1998). Design and self-assembly of two-dimensional DNA crystals. *Nature*.

[16] Mao C., Sun W., Seeman N. (1999). Designed two-dimensional DNA Holliday junction arrays visualized by atomic force microscopy. *Journal of the American Chemical Society*.

[17] Yan H., Park S. H., Finkelstein G., Reif J. H., LaBean T. H. (2003). DNA-templated self-assembly of protein arrays and highly conductive nanowires. *Science*.

[18] Williams S., Lund K., Lin C., Wonka P., Lindsay S., Yan H., Goel A., Simmel F. C., Sosík P. (2008). Tiamat: a three-dimensional editing tool for complex DNA structures. *DNA Computing. DNA 2008*.

[19] Yang Y., Han D. R., Nangreave J., Liu Y., Yan H. (2012). DNA origami with double-stranded DNA as a unified scaffold. *ACS Nano*.

[20] Woo S., Rothemund P. W. K. (2011). Erratum: programmable molecular recognition based on the geometry of DNA nanostructures. *Nature Chemistry*.

[21] Wang P. F., Gaitanaros S., Lee S., Bathe M., Shih W. M., Ke Y. (2016). Programming self-assembly of DNA origami honeycomb two-dimensional lattices and plasmonic metamaterials. *Journal of the American Chemical Society*.

[22] Wei B., Dai M., Yin P. (2012). Complex shapes self-assembled from single-stranded DNA tiles. *Nature*.

[23] Chen J., Seeman N. C. (1991). Synthesis from DNA of a molecule with the connectivity of a cube. *Nature*.

[24] Zhang Y., Seeman N. C. (1994). Construction of a DNA-truncated octahedron. *Journal of the American Chemical Society*.

[25] Goodman R. P., Schaap I. A. T., Tardin C. F. (2005). Rapid chiral assembly of rigid DNA building blocks for molecular nanofabrication. *Science*.

[26] Wang P., Wu S., Tian C. (2016). Retrosynthetic analysis-guided breaking tile symmetry for the assembly of complex DNA nanostructures. *Journal of the American Chemical Society*.

[27] Tian C., Li X., Liu Z., Jiang W., Wang G., Mao C. (2014). Directed self-assembly of DNA tiles into complex nanocages. *Angewandte Chemie International Edition*.

[28] Ke Y., Ong Luvena L., Shih William M., Yin P. (2012). Three-dimensional structures self-assembled from DNA bricks. *Science*.

[29] Shih W. M., Quispe J. D., Joyce G. F. (2004). A 1.7-kilobase single-stranded DNA that folds into a nanoscale octahedron. *Nature*.

[30] Douglas S. M., Marblestone A. H., Teerapittayanon S., Vazquez A., Church G. M., Shih W. M. (2009). Rapid prototyping of 3D DNA-origami shapes with caDNAno. *Nucleic Acids Research*.

[31] Veneziano R., Ratanalert S., Zhang K. M. (2016). Designer nanoscale DNA assemblies programmed from the top down. *Science*.

[32] Jun H., Shepherd T. R., Zhang K. (2019). Automated sequence design of 3D polyhedral wireframe DNA origami with honeycomb edges. *ACS Nano*.

[33] Andersen E. S., Dong M., Nielsen M. M. (2009). Self-assembly of a nanoscale DNA box with a controllable lid. *Nature*.

[34] Sun W., Boulais E., Hakobyan Y. (2014). Casting inorganic structures with DNA molds. *Science*.

[35] Helmi S., Ziegler C., Kauert D. J., Seidel R. (2014). Shape-controlled synthesis of gold nanostructures using DNA origami molds. *Nano Letters*.

[36] Ye J., Aftenieva O., Bayrak T. (2021). Complex metal nanostructures with programmable shapes from simple DNA building blocks. *Advanced Materials*.

[37] Ye J. J., Weichelt R., Kemper U. (2020). Casting of gold nanoparticles with high aspect ratios inside DNA molds. *Small*.

[38] Ye J., Helmi S., Teske J., Seidel R. (2019). Fabrication of metal nanostructures with programmable length and patterns using a modular DNA platform. *Nano Letters*.

[39] Weichelt R., Ye J. J., Banin R., Eychmuller A., Seidel R. (2019). DNA-mediated self-assembly and metallization of semiconductor nanorods for the fabrication of nanoelectronic interfaces. *Chemistry-a European Journal*.

[40] Bayrak T., Helmi S., Ye J. (2018). DNA-mold templated assembly of conductive gold nanowires. *Nano Letters*.

[41] Benson E., Mohammed A., Gardell J. (2015). DNA rendering of polyhedral meshes at the nanoscale. *Nature*.

[42] Poppleton E., Bohlin J., Matthies M., Sharma S., Zhang F., Šulc P. (2020). Design, optimization and analysis of large DNA and RNA nanostructures through interactive visualization, editing and molecular simulation. *Nucleic Acids Research*.

[43] Tikhomirov G., Petersen P., Qian L. (2018). Triangular DNA origami tilings. *Journal of the American Chemical Society*.

[44] Zhang T., Hartl C., Frank K. (2018). 3D DNA origami crystals. *Advanced Materials*.

[45] Ji M., Liu J. L., Dai L. Z., Wang L., Tian Y. (2020). Programmable cocrystallization of DNA origami shapes. *Journal of the American Chemical Society*.

[46] Minev D., Wintersinger C. M., Ershova A., Shih W. M. (2021). Robust nucleation control via crisscross polymerization of highly coordinated DNA slats. *Nature Communications*.

[47] Chen Z., Liu C., Cao F., Ren J., Qu X. (2018). DNA metallization: principles, methods, structures, and applications. *Chemical Society Reviews*.

[48] Braun E., Eichen Y., Sivan U., Ben-Yoseph G. (1998). DNA-templated assembly and electrode attachment of a conducting silver wire. *Nature*.

[49] Watson S. M., Mohamed H. D., Horrocks B. R., Houlton A. (2013). Electrically conductive magnetic nanowires using an electrochemical DNA-templating route. *Nanoscale*.

[50] Jayaraman S., Tang W., Yongsunthon R. (2011). Electrochemical synthesis of M:DNA nanohybrids. *Journal of the Electrochemical Society*.

[51] Mohamed H. D. A., Watson S. M. D., Horrocks B. R., Houlton A. (2015). Chemical and electrochemical routes to DNA-templated rhodium nanowires. *Journal of Materials Chemistry C*.

[52] Kundu S., Wang K., Huitink D., Liang H. (2009). Photoinduced formation of electrically conductive thin palladium nanowires on DNA scaffolds. *Langmuir*.

[53] Berti L., Alessandrini A., Facci P. (2005). DNA-templated photoinduced silver deposition. *Journal of the American Chemical Society*.

[54] Erler C., Guenther K., Mertig M. (2009). Photo-induced synthesis of DNA-templated metallic nanowires and their integration into micro-fabricated contact arrays. *Applied Surface Science*.

[55] Guo W., Yuan J., Wang E. (2009). Oligonucleotide-stabilized Ag nanoclusters as novel fluorescence probes for the highly selective and sensitive detection of the Hg^2+^ ion. *Chemical Communications*.

[56] Chandler M., Shevchenko O., Vivero-Escoto J. L., Striplin C. D., Afonin K. A. (2020). DNA-templated synthesis of fluorescent silver nanoclusters. *Journal of Chemical Education*.

[57] Zhang J., Wang X., Fu Y. (2013). Highly active subnano palladium clusters embedded in i-motif DNA. *Langmuir*.

[58] Park J., Song J., Park J., Park N., Kim S. (2014). Branched DNA-based synthesis of fluorescent silver nanocluster. *Bulletin of the Korean Chemical Society*.

[59] Wang Z.-G., Liu Q., Li N., Ding B. (2016). DNA-based nanotemplate directed in situ synthesis of silver nanoclusters with specific fluorescent emission: surface-guided chemical reactions. *Chemistry of Materials*.

[60] Basu S., Jana S., Pande S., Pal T. (2008). Interaction of DNA bases with silver nanoparticles: assembly quantified through SPRS and SERS. *Journal of Colloid and Interface Science*.

[61] Gourishankar A., Shukla S., Ganesh K. N., Sastry M. (2004). Isothermal titration calorimetry studies on the binding of DNA bases and PNA base monomers to gold nanoparticles. *Journal of the American Chemical Society*.

[62] Wang Z., Tang L., Tan L. H., Li J., Lu Y. (2012). Discovery of the DNA “genetic code” for abiological gold nanoparticle morphologies. *Angewandte Chemie International Edition*.

[63] Li J., Zhu Z., Liu F. (2016). DNA-mediated morphological control of silver nanoparticles. *Small*.

[64] Wei Z., Yu Y., Hu S., Yi X., Wang J. (2021). Bifunctional diblock DNA-mediated synthesis of nanoflower-shaped photothermal nanozymes for a highly sensitive colorimetric assay of cancer cells. *ACS Applied Materials & Interfaces*.

[65] Satyavolu N. S., Tan L. H., Lu Y. (2016). DNA-mediated morphological control of Pd-Au bimetallic nanoparticles. *Journal of the American Chemical Society*.

[66] Ma X., Huh J., Park W., Lee L. P., Kwon Y. J., Sim S. J. (2016). Gold nanocrystals with DNA-directed morphologies. *Nature Communications*.

[67] Ma X., Song S., Kim S. (2019). Single gold-bridged nanoprobes for identification of single point DNA mutations. *Nature Communications*.

[68] Bayrak T., Jagtap N. S., Erbe A. (2018). Review of the electrical characterization of metallic nanowires on DNA templates. *International Journal of Molecular Sciences*.

[69] Ranasinghe D. R., Aryal B. R., Westover T. R. (2020). Seeding, plating and electrical characterization of gold nanowires formed on self-assembled DNA nanotubes. *Molecules*.

[70] Kim K.-I., Lee S., Jin X., Kim S. J., Jo K., Lee J. H. (2017). DNA binding peptide directed synthesis of continuous DNA nanowires for analysis of large DNA molecules by scanning electron microscope. *Small*.

[71] Eidelshtein G., Fardian-Melamed N., Gutkin V. (2016). Synthesis and properties of novel silver-containing DNA molecules. *Advanced Materials*.

[72] Stern A., Eidelshtein G., Zhuravel R. (2018). Highly conductive thin uniform gold-coated DNA nanowires. *Advanced Materials*.

[73] Keren K., Krueger M., Gilad R., Ben-Yoseph G., Sivan U., Braun E. (2002). Sequence-specific molecular lithography on single DNA molecules. *Science*.

[74] Keren K., Berman R. S., Buchstab E., Sivan U., Braun E. (2003). DNA-templated carbon nanotube field-effect transistor. *Science*.

[75] Keren K., Berman R. S., Braun E. (2004). Patterned DNA metallization by sequence-specific localization of a reducing agent. *Nano Letters*.

[76] Westover T. R., Aryal B. R., Ranasinghe D. R. (2020). Impact of polymer-constrained annealing on the properties of DNA origami-templated gold nanowires. *Langmuir*.

[77] Stanca S. E., Eritja R., Fitzmaurice D. (2006). DNA-templated assembly of nanoscale architectures for next-generation electronic devices. *Faraday Discussions*.

[78] Wang G. Q., Ishikawa A., Eguchi A. (2012). Sequence-specific metallization of single divalent DNA–nanoparticle conjugates: a potential route to single-electron devices. *ChemPlusChem*.

[79] Rudiuk S., Venancio-Marques A., Hallais G., Baigl D. (2013). Preparation of one- to four-branch silver nanostructures of various sizes by metallization of hybrid DNA-protein assemblies. *Soft Matter*.

[80] Schreiber R., Kempter S., Holler S. (2011). DNA origami-templated growth of arbitrarily shaped metal nanoparticles. *Small*.

[81] Liu J., Geng Y., Pound E. (2011). Metallization of branched DNA origami for nanoelectronic circuit fabrication. *ACS Nano*.

[82] Geng Y., Liu J., Pound E., Gyawali S., Harb J. N., Woolley A. T. (2011). Rapid metallization of lambda DNA and DNA origami using a Pd seeding method. *Journal of Materials Chemistry*.

[83] Geng Y., Pearson A. C., Gates E. P. (2013). Electrically conductive gold- and copper-metallized DNA origami nanostructures. *Langmuir*.

[84] Teschome B., Facsko S., Schönherr T., Kerbusch J., Keller A., Erbe A. (2016). Temperature-dependent charge transport through individually contacted DNA origami-based Au nanowires. *Langmuir*.

[85] Schreiber R., Santiago I., Ardavan A., Turberfield A. J. (2016). Ordering gold nanoparticles with DNA origami nanoflowers. *ACS Nano*.

[86] Pilo-Pais M., Goldberg S., Samano E., Labean T. H., Finkelstein G. (2011). Connecting the nanodots: programmable nanofabrication of fused metal shapes on DNA templates. *Nano Letters*.

[87] Pearson A. C., Liu J., Pound E. (2012). DNA origami metallized site specifically to form electrically conductive nanowires. *The Journal of Physical Chemistry B*.

[88] Uprety B., Gates E. P., Geng Y., Woolley A. T., Harb J. N. (2014). Site-specific metallization of multiple metals on a single DNA origami template. *Langmuir*.

[89] Luo X., Lachance-Brais C., Bantle A., Sleiman H. F. (2020). The assemble, grow and lift-off (AGLO) strategy to construct complex gold nanostructures with pre-designed morphologies. *Chemical Science*.

[90] Bayrak T., Martinez-Reyes A., Arce D. D. R., Kelling J., Samano E. C., Erbe A. (2021). Fabrication and temperature-dependent electrical characterization of a C-shape nanowire patterned by a DNA origami. *Scientific Reports*.

[91] Uprety B., Jensen J., Aryal B. R., Davis R. C., Woolley A. T., Harb J. N. (2017). Directional growth of DNA-functionalized nanorods to enable continuous, site-specific metallization of DNA origami templates. *Langmuir*.

[92] Aryal B. R., Ranasinghe D. R., Westover T. R. (2020). DNA origami mediated electrically connected metal-semiconductor junctions. *Nano Research*.

[93] Pal S., Varghese R., Deng Z. (2011). Site-specific synthesis and in situ immobilization of fluorescent silver nanoclusters on DNA nanoscaffolds by use of the Tollens reaction. *Angewandte Chemie International Edition*.

[94] Roy S., Olesiak M., Shang S., Caruthers M. H. (2013). Silver nanoassemblies constructed from boranephosphonate DNA. *Journal of the American Chemical Society*.

[95] Li N., Shang Y., Xu R. (2019). Precise organization of metal and metal oxide nanoclusters into arbitrary patterns on DNA origami. *Journal of the American Chemical Society*.

[96] Zhao Y. M., Zhang C., Yang L. L. (2021). Programmable and site-specific patterning on DNA origami templates with heterogeneous condensation of silver and silica. *Small*.

[97] Jia S., Wang J., Xie M. (2019). Programming DNA origami patterning with non-canonical DNA-based metallization reactions. *Nature Communications*.

[98] Zhang Y., Qu Z. B., Jiang C. (2021). Prescribing silver chirality with DNA origami. *Journal of the American Chemical Society*.

[99] Deng Z., Mao C. (2004). Molecular lithography with DNA nanostructures. *Angewandte Chemie International Edition*.

[100] Shen B. X., Linko V., Tapio K., Kostiainen M. A., Toppari J. J. (2015). Custom-shaped metal nanostructures based on DNA origami silhouettes. *Nanoscale*.

[101] Shen B., Linko V., Tapio K. (2018). Plasmonic nanostructures through DNA-assisted lithography. *Science Advances*.

[102] Piskunen P., Shen B., Keller A., Toppari J. J., Kostiainen M. A., Linko V. (2021). Biotemplated lithography of inorganic nanostructures (BLIN) for versatile patterning of functional materials. *ACS Applied Nano Materials*.

[103] Barreda J. L., Hu L., Yu L. (2020). Controlled fabrication of DNA molecular templates for in situ formation and measurement of ultrathin metal nanostructures. *Nano Letters*.

[104] Narayan N., Meiyazhagan A., Vajtai R. (2019). Metal nanoparticles as green catalysts. *Materials*.

[105] Zinchenko A., Miwa Y., Lopatina L. I., Sergeyev V. G., Murata S. (2014). DNA hydrogel as a template for synthesis of ultrasmall gold nanoparticles for catalytic applications. *ACS Applied Materials & Interfaces*.

[106] Zinchenko A., Che Y., Taniguchi S., Lopatina L. I., Sergeyev V. G., Murata S. (2016). Metallization of DNA hydrogel: application of soft matter host for preparation and nesting of catalytic nanoparticles. *Journal of Nanoparticle Research*.

[107] Li W., Liu L., Fu Y., Sun Y., Zhang J., Zhang R. (2013). Effects of polymorphic DNA on the fluorescent properties of silver nanoclusters. *Photochemical & Photobiological Sciences*.

[108] Fu Y., Zhao X., Zhang J., Li W. (2014). DNA-based platinum nanozymes for peroxidase mimetics. *The Journal of Physical Chemistry C*.

[109] Hao L. L., Zhao L. Y., Li G. B. (2020). Ultrasensitive detection of CYFRA 21-1 DNA via SI-RAFT based in-situ metallization signal amplification. *Microchemical Journal*.

[110] Wu L., Wang J. S., Ren J. S., Qu X. G. (2014). Ultrasensitive telomerase activity detection in circulating tumor cells based on DNA metallization and sharp solidstate electrochemical techniques. *Advanced Functional Materials*.

[111] Liu G., Shao Y., Peng J. (2013). Highly thymine-dependent formation of fluorescent copper nanoparticles templated by ss-DNA. *Nanotechnology*.

[112] Chen J., Liu J., Fang Z., Zeng L. (2012). Random dsDNA-templated formation of copper nanoparticles as novel fluorescence probes for label-free lead ions detection. *Chemical Communications*.

[113] Gong L., Kuai H., Ren S. (2015). Ag nanocluster-based label-free catalytic and molecular beacons for amplified biosensing. *Chemical Communications*.

[114] Bowden M., Heldebrant D. J., Karkamkar A., Proffen T., Schenter G. K., Autrey T. (2010). The diammoniate of diborane: crystal structure and hydrogen release. *Chemical Communications*.

[115] Al-Hinai M. N., Hassanien R., Wright N. G., Horsfall A. B., Houlton A., Horrocks B. R. (2013). Networks of DNA-templated palladium nanowires: structural and electrical characterisation and their use as hydrogen gas sensors. *Faraday Discussions*.

[116] Zhao K., Chang Q., Chen X., Zhang B., Liu J. (2009). Synthesis and application of DNA-templated silver nanowires for ammonia gas sensing. *Materials Science and Engineering: C*.

[117] Lu J., Yang L., Xie A., Shen Y. (2009). DNA-templated photo-induced silver nanowires: fabrication and use in detection of relative humidity. *Biophysical Chemistry*.

[118] Lim J. Y. C., Yu Y., Jin G. (2020). Establishing empirical design rules of nucleic acid templates for the synthesis of silver nanoclusters with tunable photoluminescence and functionalities towards targeted bioimaging applications. *Nanoscale Advances*.

[119] Han G. M., Jia Z. Z., Zhu Y. J., Jiao J. J., Kong D. M., Feng X. Z. (2016). Biostable L-DNA-templated aptamer-silver nanoclusters for cell-type-specific imaging at physiological temperature. *Analytical Chemistry*.

